# A pilot crossover trial assessing the exercise performance patients with chronic obstructive pulmonary disease

**DOI:** 10.1038/s41598-022-07698-z

**Published:** 2022-03-09

**Authors:** Ke-Yun Chao, Wei-Lun Liu, Yasser Nassef, Pin-Zhen Lai, Jong-Shyan Wang

**Affiliations:** 1 Department of Respiratory Therapy, Fu Jen Catholic University Hospital, Fu Jen Catholic University, New Taipei City, Taiwan; 2 School of Physical Therapy, Graduate Institute of Rehabilitation Sciences, Chang Gung University, Taoyuan, Taiwan; 3 Department of Emergency and Critical Care Medicine, Fu Jen Catholic University Hospital, Fu Jen Catholic University, New Taipei City, Taiwan; 4 School of Medicine, College of Medicine, Fu Jen Catholic University, New Taipei City, Taiwan; 5 Institution of Medicine, Chung Shan Medical University, Taizhong, Taiwan; 6 Department of Physical Medicine and Rehabilitation, Keelung Chang Gung Memorial Hospital, Keelung, Taiwan; 7 Department of Physical Therapy, School of Physical Therapy, College of Medicine, Graduate Institute of Rehabilitation Science, Chang Gung University, No. 259, Wenhua 1st Rd., Guishan Dist., Taoyüan, 33302 Taiwan; 8 Research Center for Chinese Herbal Medicine, College of Human Ecology, Chang Gung University of Science and Technology, Taoyuan, Taiwan

**Keywords:** Clinical trial design, Therapeutics

## Abstract

Noninvasive ventilation improves exercise performance in patients with chronic obstructive pulmonary disease (COPD). However, the effect of helmet ventilation (HV) on the short-term self-paced exercise performance of patients with COPD remains unclear. This study investigated the use of HV during a 6 min walk test (6MWT) and analyzed its short-term cardiopulmonary outcomes in patients with stable COPD. A single-site crossover trial was conducted in a pulmonary rehabilitation outpatient department. A total of 20 stable patients with COPD without disability were enrolled. The participants performed 6MWTs with and without HV on two consecutive days. The outcome measures were the distance walked in the 6MWT and the physiological and cardiopulmonary parameters. The mean difference in meters walked between the HV-aided and unaided walk tests was 15.4 ± 37.2 (95% confidence interval: − 2.03 to 32.8 m; *p* = .145). During the 6MWT, the peak heart rate was significantly higher when walking was aided by HV than when it was unaided (*p* < .001). The energy expenditure index, walking speed, oxygen saturation nadir, and hemodynamic parameters were comparable. Although carbon dioxide levels inside the helmet increased after the walk test, the participants’ transcutaneous carbon dioxide measurements remained unchanged. HV did not improve the short-term self-paced exercise performance in patients with stable mild-to-moderate COPD. Further research should focus on noninvasive ventilation delivered via helmets in exercise training to determine the setting strategy, breathing circuit configuration, and effects of regular exercise.

ClinicalTrial.gov: NCT04156724; IRB number: C108032.

## Introduction

People with chronic obstructive pulmonary disease (COPD) often experience physical inactivity and exercise intolerance caused by muscular weakness and dyspnea^1,2^. According to a joint American Thoracic Society (ATS) and European Respiratory Society statement, pulmonary rehabilitation is “an evidence-based, multidisciplinary, and comprehensive intervention for patients with chronic respiratory diseases who are symptomatic and often have decreased daily life activities^3^.” Pulmonary rehabilitation has been demonstrated to improve clinical outcomes and exercise capacity in patients with symptomatic COPD^4–6^.

Exercise training is a critical component of pulmonary rehabilitation. This is supported by evidence demonstrating that it is an effective therapeutic intervention in the management of COPD^7,8^. However, for some individuals, performing exercise training at an adequate intensity is difficult because of their physical condition^9^. The use of noninvasive ventilation (NIV) has been suggested as an adjunct to an exercise program that allows patients to exercise at a higher intensity and improve their exercise intolerance and health-related quality of life^10–13^. However, the use of NIV is complex and labor-intensive and may only be feasible in specialist units and for patients who have experience with this treatment^3,14^. Studies have reported dropout rates of 7.1–42% from exercise training with adjunct NIV^12,15–17^. NIV is usually delivered via an oronasal facemask^18,19^, and nasal bridge ulceration, skin breakdown, air leaks, and discomfort are the most common complications in patients using NIV^20–22^.

The helmet used in this study came in the form of a transparent plastic hood, which was originally designed for hyperbaric oxygen therapy^23^. Instead of having seals around the nose and mouth, the helmet surrounds the patient’s head and is sealed around the neck by a soft collar. The helmet is currently being introduced as an alternative interface for oronasal facemasks^24,25^. Compared with facemasks, the delivery of NIV via a helmet has several advantages, including improved tolerability, fewer air leaks, and improved seal integrity at the neck^26,27^. Therefore, the helmet’s design may allow patients with COPD to exercise without intolerance. This could increase exercise performance and enable more patients with COPD to benefit from NIV.

To the best of our knowledge, no study has evaluated helmet ventilation (HV) for exercise training. The aim of the present study was to investigate whether HV increased short-term self-paced exercise performance and to analyze cardiopulmonary outcomes.

## Results

**Figure 1:**
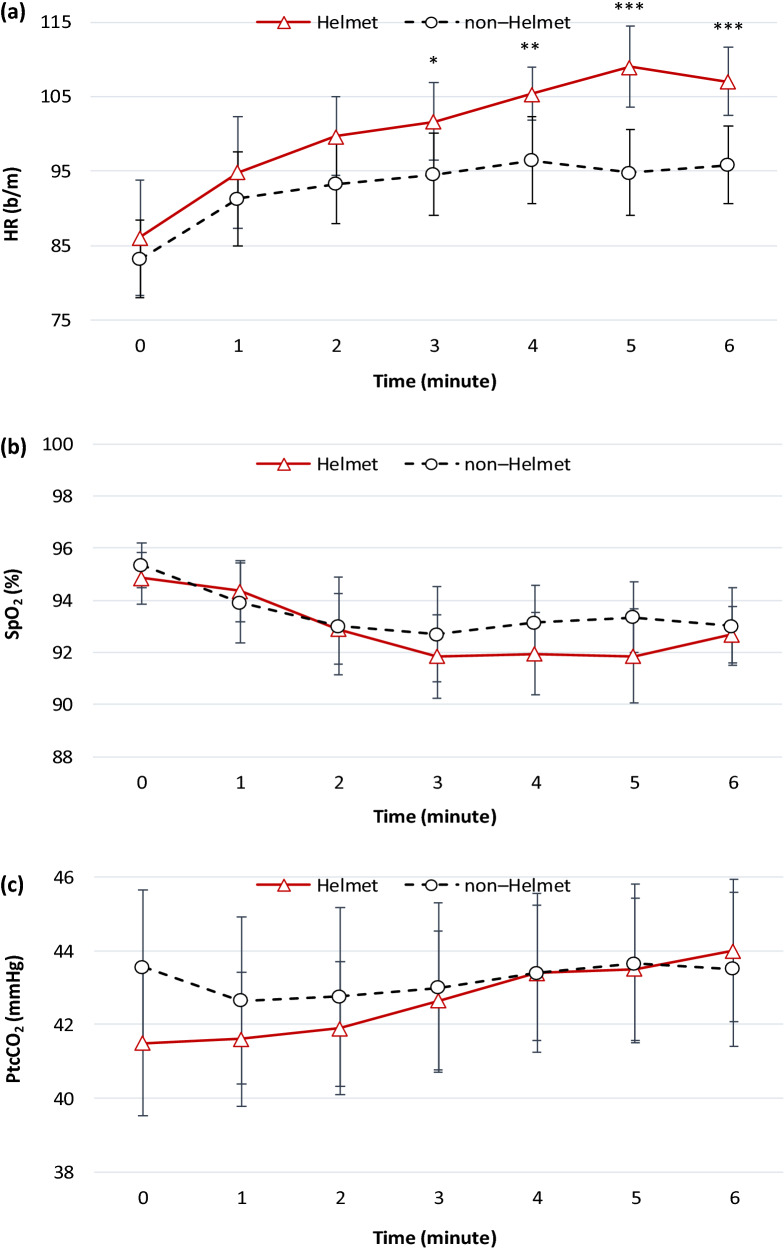
Physiological trends for (**a**) heart rate (HR), (**b**) oxygen saturation (SpO_2_), and (**c**) transcutaneous carbon dioxide tension (PtcCO_2_) during the 6-min walk test. ^*^p < .05, ^**^p < .01, ^***^p < .001.

In total, 20 participants without disability were enrolled in the present study, and no participants dropped out. The baseline demographic characteristics are provided in Table 1. No major adverse events or claustrophobia were detected during the walk test, although three participants required additional oxygen support because of low oxygen saturation (SpO_2_) during the HV-aided walk. The mean difference in 6MWD between the HV-aided and unaided walk was 15.4 ± 37.2 (95% confidence interval: − 2.03–32.8 m; *p* = 0.145; Table 2). The baseline parameters of the participants in the HV-aided and unaided walk were comparable. During the 6-min walk test (6MWT), the peak heart rate (HR) was significantly higher when walking was aided by HV than when it was unaided (median: 115 b/m [IQR: 102–125] b/m vs. median: 100 b/m [IQR: 89.5–113] b/m, *p* < 0.001); walking speed, SpO_2_ nadir, and energy expenditure index (EEI) were comparable. After the 6MWT, the HR and respiratory rate were higher during the HV-aided walk than during the unaided walk. A significant difference was observed in HR during the 6MWT, but the changes in SpO_2_ and transcutaneous carbon dioxide tension (PtcCO_2_) were not statistically significant (Fig. 1). The hemodynamic parameters following the HV-aided and unaided 6MWT were comparable (Table 3). The peak inspiratory pressure flow rate was higher than 100 L/min, and the maximum carbon dioxide (CO_2_) tension air leaks from the helmet increased after the walk test (Table 4).

**Table 1:** Participant characteristics.

Subjects		20
**Demographic data**		
Gender (male/female)	17/3	
Age (years)	70.5	(63.3–77)
BMI (kg/m^2^)	23.5	(21.7–27.1)
Former smoker (%)	16	(80)
**Lung function**		
FEV_1_, % predicted	71.5	(62.3–90.5)
FVC, % predicted	93	(78.8–96.8)
FEV_1_/FVC, %	66	(53.5–68)
FRC, % predicted	131	(113–153)
RV, % predicted	127	(115–159)
TLC, % predicted	130	(109–143)
**GOLD stage**		
Stage I, (%)	6	(30)
Stage II, (%)	12	(60)
Stage III, (%)	2	(10)

Data are presented as median (IQR) or number (%).

*BMI* body mass index, *FEV*_*1*_ forced expiratory volume in the first second, *FVC* forced vital capacity, *FRC*, functional residual capacity, *RV* residual volume, *TLC* total lung capacity, *GOLD* Global Initiative for Chronic Obstructive Lung Disease.

**Table 2:** Physiological and 6MWT outcomes.

	Helmet (n = 20)		Non-Helmet (n = 20)		Mean change (Helmet minus non-Helmet)			*p*-value
							95% CI	
**6MWT outcome**								
6WMD, m	346	(321–415)	331	(279–419)	15.4	± 37.2	− 2.03–32.8	0.145
Walking speed, m/min	57.6	(53.5–69.2)	55.2	(46.5–69.8)	2.57	± 6.2	− 0.34–5.47	0.145
HR peak, b/m	115	(102–125)	100	(89.5–113)	12.8	± 13.9	6.23–19.3	< 0.001***
SpO_2_ nadir, %	92	(90–93.8)	92	(90–94)	− 0.65	± 3.6	− 2.34–1.04	0.434
EEI, beat/meter walked	1.79	(1.41–1.96)	1.67	(1.45–1.96)	0.06	± 0.35	− 0.10–0.23	0.433
**Before 6MWT**								
HR, b/m	84.5	(70.8–102)	80	(72–97.5)	7.6	± 11.9	2.05–13.2	0.384
SpO_2_, %	95.5	(94–96.8)	96	(94–97)	− 0.4	± 1.6	− 1.15–0.35	0.255
RR, b/m	16	(15–17)	16	(15–16.8)	0.2	± 0.7	− 0.13–0.53	0.206
Borg-D	0	(0–0.75)	0	(0–0)				0.542
sBP, mmHg	125	(114–137)	126	(115–135)	− 3.25	± 16.7	− 11.1–4.56	0.384
dBP, mmHg	80.5	(66–86)	79.5	(68.3–86)	− 0.05	± 7.79	− 3.7–3.6	0.831
MAP, mmHg	95.7	(81.6–101)	95.3	(87.5–99.4)	− 1.12	± 9.47	− 5.55–3.32	0.616
PtcCO_2_, mmHg	41.5	(38–44)	43.5	(39–45)	− 1.9	± 4.35	− 3.94–0.14	0.101
**After 6MWT**								
HR, b/m	104	(98.5–114)	93.5	(83.5–107)	9.2	± 13.6	2.81–15.6	0.002**
SpO_2_, %	93	(91.3–94.8)	94	(93–95.8)	− 0.3	± 3.28	− 1.83–1.23	0.346
RR, b/m	20	(18.3–22)	18	(17.3–19)	1.85	± 2.43	0.71–2.99	0.004**
Borg-D	3	(1–5)	2	(0.25–3)				0.107
sBP, mmHg	152	(132–165)	142	(127–155)	9.25	± 32.9	− 6.14–24.6	0.198
dBP, mmHg	84.5	(73–94)	86	(71.3–93.5)	0.05	± 12.2	− 5.64–5.74	0.466
MAP, mmHg	109	(90.8–115)	103	(91.8–116)	3.12	± 17.4	− 5.01–11.2	0.332
PtcCO_2_, mmHg	44	(41.3–46)	43	(41–46)	0.6	± 4.75	− 1.62–2.82	0.613

Data are presented as mean ± SD or median (IQR).

**p < .01; ***p < .001.

*6MWT* 6-min walk test, *6WMD* 6-min walk distance, *HR* heart rate, *SpO*_*2*_ oxygen saturation, *EEI* energy expenditure index, *RR* respiratory rate, *Borg-D* Borg dyspnea score, *sBP* systolic blood pressure, *dBP* diastolic blood pressure, *MAP* mean arterial pressure, *PtcCO*_*2*_ transcutaneous carbon dioxide tension.

**Table 3:** Hemodynamic outcomes.

	Helmet (n = 20)		Non-Helmet (n = 20)		Mean change (Helmet minus non–Helmet)			*p*-value
							95% CI	
**Before 6MWT**								
SV, ml	51.7	(46.1–59.1)	54	(48.9–63.1)	− 1.88	± 4.66	− 4.06 to 0.3	0.052
CO, L/min	4.29	(3.74–4.81)	3.92	(3.56–4.87)	− 0.004	± 0.65	− 0.31 to 0.3	0.881
ICON, unit	33	(29.9–44.5)	38.3	(33.7–46.1)	− 2.26	± 6.6	− 5.35 to 0.83	0.108
FTC, ms	321	(311–330)	324	(317–328)	− 2.42	± 6.55	− 5.48 to 0.65	0.093
SVV, %	14.2	(11.5–17.1)	16.8	(11.4–21)	− 2.16	± 5.9	− 4.93 to 0.6	0.153
SVR, dynes · sec/cm^5^/m^2^	1785	(1581–1972)	1787	(1353–2127)	− 63.2	± 387	− 244 to 118	0.627
SVRI, unit	2959	(2611–3362)	2939	(2448–3583)	− 92	± 632	− 388 to 204	0.627
STR, unit	0.45	(0.41–0.54)	0.47	(0.42–0.52)	0.01	± 0.06	− 0.02 to 0.04	0.736
PEP, ms	129	(122–140)	132	(120–141)	− 1.96	± 13.3	− 8.18 to 4.25	0.654
LVET, ms	283	(262–301)	289	(272–301)	− 6.52	± 22.3	− 16.9 to 3.91	0.287
**After 6MWT**								
SV, ml	58.7	(50.8–66.4)	57.3	(55–65.2)	− 1.01	± 8.25	− 4.87 to 2.86	0.926
CO, L/min	5	(4.66–5.81)	4.85	(4.07–5.54)	0.14	± 1.12	− 0.39 to 0.66	0.247
ICON, unit	47.5	(35.5–52.6)	43.8	(31.5–58.4)	− 1.72	± 13.1	− 7.85 to 4.41	0.926
FTC, ms	325	(321–333)	329	(321–336)	− 0.96	± 12.2	− 6.67 to 4.75	0.681
SVV, %	18.3	(15.1–20.3)	16.4	(12.6–20.3)	2.44	± 5.52	− 0.15 to 5.02	0.076
SVR, dynes · sec/cm^5^/m^2^	1447	(1320–1618)	1681	(1160–1922)	− 107	± 375	− 283 to 67.9	0.191
SVRI, unit	2484	(2105–2849)	2836	(1899–3310)	− 167	± 622	− 458 to 124	0.204
STR, unit	0.44	(0.37–0.54)	0.45	(0.4–0.5)	− 0.003	± 0.09	− 0.04 to 0.04	0.708
PEP, ms	117	(108–137)	125	(110–134)	− 4.84	± 16.3	− 12.5 to 2.82	0.135
LVET, ms	275	(250–292)	284	(269–301)	− 7.36	± 23.5	− 18.4 to 3.65	0.225

Data are presented as mean ± SD or median (IQR).

*SV* stroke volume, *CO* cardiac output, *ICON* index of contractility, *FTC* correct flow time, *SVV* stroke volume variation, *SVR* systemic vascular resistance, *SVRI* systemic vascular resistance index, *STR* systolic time ratio, *PEP* pre-ejection period, *LVET* left ventricular ejection time.

**Table 4:** Ventilator parameters and measured maximum carbon dioxide tension.

	Beginning of test (n = 20)		End of test (n = 20)		Mean change (Value at end of test minus at beginning of test)			*p*-value
							95% CI	
Flow rate^a^, L/min	121	(108–135)	114	(107–125)	− 4.71	± 23.5	− 15.7–6.31	0.382
Air Leaks, L/min	48.9	(34.7–59.6)	52.6	(37.2–78.9)	13.9	± 26.6	1.5–26.4	0.03*
CO_2_ Max, mmHg	7.5	(6–11)	11.5	(8–14.8)	3.3	± 3.08	1.86–4.74	< 0.001***

Data are presented as mean ± SD or median (IQR). *p < .05; ***p < .001.

^a^Measured when the flow rate was at the peak inspiratory pressure; CO_2_ Max: maximum carbon dioxide tension.

## Discussion

In patients with stable COPD, the use of HV during the walk test did not affect the walk distance. Although the HR peak during the test and HR after the test was higher when the participants were aided by HV than when they were unaided, the EEI was comparable in both scenarios and HV did not affect the walking economy. Dreher et al.^28^ applied NIV via a facemask during the walk test to patients with very severe COPD. The use of NIV improved oxygenation and dyspnea and increased the 6MWD. Despite the insufficient statistical power in their results, a subgroup analysis was performed to assess the primary and secondary outcomes according to the COPD stages (mild or moderate-to-severe). Among the 14 participants with moderate-to-severe COPD, the statistically significant results for the primary and secondary outcomes were consistent compared with the whole group (see Supplementary Table S1 online). However, for the six participants with mild COPD, the results were comparable between the HV-aided and unaided 6MWT (see Supplementary Table S2 online).

In the present study, the maximum CO_2_ tension inside the helmet increased after the walk test, but no significant difference in PtcCO_2_ was observed between the HV-aided walk and the unaided walk. Although the modality of CO_2_ measurement inside the helmet in this study was distinct from that of previous studies, similar results were obtained^29,30^. Increased maximum CO_2_ tension inside the helmet might suggest insufficient CO_2_ elimination during the test. Because the 6MWT is a self-paced exercise^31^, the ability to remove CO_2_ might worsen when the intensity of the exercise increases. The use of NIV during exercise training improves exercise capacity and ameliorates the physiological impact, and it may help patients with COPD escalate their training intensity^12^. Because NIV requires a tight-fitting facemask, intolerance is the most common reason for patients with COPD to stop exercise training. Although the effect of withdrawing from NIV-aided or unaided exercise training on these patients is unclear, notable dropout rates, ranging from 21 to 42%, have been reported in previous studies^12^. As an interface for NIV, the helmet has higher tolerability^26,27^, and no participants withdrew from the present study. Physical activity or exercise reduces the integrity of the seal that separates the patient and the NIV interface, but the helmet improves seal integrity, resulting in fewer air leaks from the interface^26,27^. In the present study, the air leaks increased after the walk test without affecting the flow rate at peak inspiratory pressure. Although claustrophobia is the most frequently mentioned complication of HV, and is possibly unavoidable, it is not commonly reported in clinical cases^24,32^. Because the 6MWT is a submaximal exercise assessment, no significant change was observed in the hemodynamic outcomes of this study.

The use of HV in exercise training is a relatively novel approach to NIV, and the present study had several limitations. First, the NIV settings were based on a previous study by Patel et al.^24^ in which the effectiveness of HV in patients with severe acute respiratory distress syndrome was investigated. In patients with COPD, the minute ventilation when resting was no different between acute and chronic respiratory failure status, and it was similar to that of healthy participants^33^. However, minute ventilation differed between patients with COPD when exercising and patients with acute respiratory failure when resting on their beds. During exercise, the minute ventilation of healthy participants might increase from resting values of approximately 5–6 L/min to more than 100 L/min^34,35^; this increase, however, goes to 30–40 L/min in patients with COPD^36^. Increasing the ventilation setting may achieve different results. Second, the single-limb circuit used in the present study was restricted to the noninvasive ventilator. An in-vitro study demonstrated that compared with the standard dual-limb circuit with a Y-piece, a double-tube circuit connected to the helmet had shorter inspiratory and expiratory delays, a longer synchrony time, and no wasted effort, which resulted in a better patient–ventilator interaction^37^. This therefore suggests a difference between a single-limb circuit and double-tube circuit when using HV. In a single-limb circuit with a facemask, both inspiratory and expiratory gas flow passes through the same limb, but it comes with the risk of CO_2_ rebreathing^38^. Third, the 6MWT only reflects functional capacity^39^; the effect of HV on high-intensity exercise training is unclear. Further research into exercise capacity tests for HV in exercise training should be conducted using a cycle ergometer^40,41^ or treadmill^42,43^ to clarify the long-term effects. This is the first study to investigate the effects of HV on exercise training. Because no data from previous studies were available, a submaximal exercise assessment was selected for the pilot study to investigate the feasibility of HV in exercise training. The present study’s results provide a basis for future studies.

In conclusion, this pilot study demonstrated that using a helmet with a single-limb noninvasive ventilator did not improve the short-term self-paced exercise performance in patients with mild-to-moderate COPD with a ventilator setting strategy for patients with respiratory failure. The application of NIV delivered via the helmet during exercise training for pulmonary rehabilitation warrants further research with long-term follow-up to determine the setting strategy, breathing circuit configuration, and effects of regular exercise training.

## Material and methods

### Study design

This single-site crossover trial was conducted between July 2020 and January 2021 at the pulmonary rehabilitation outpatient department of Fu Jen Catholic University Hospital, Northern Taiwan. The study was approved by the Institutional Review Board, Fu Jen Catholic University, New Taipei City, Taiwan (C108032), and is registered with ClinicalTrials.gov (NCT04156724 07/11/2019). Informed written consent was obtained from all the participants.

### Participants

The diagnosis of COPD was confirmed by a pulmonary lung function test, and those who had participated in the pulmonary rehabilitation program for at least 1 month were eligible for enrollment. The COPD stage classifications used in this study were adapted from the Global Initiative for Chronic Obstructive Lung Disease 2021 report^44^. Patients were excluded if they had experienced acute COPD exacerbation within 3 months of the study, had received a diagnosis of neuromuscular disease, had an artificial airway, required mechanical ventilator or NIV support, or were unable to perform the 6MWT.

### Helmet ventilation

HV was provided through helmets of two sizes, medium and large (StarMED CaStar-R, Intersurgical, Wokingham, Berkshire, UK). The helmet is a transparent latex-free polyvinyl chloride hood that surrounds the patient’s head and seals around the neck using a soft latex-free polyurethane collar (see Supplementary Fig. S1 online). The helmet is connected to a hard plastic ring and secured to the patient with padded armpit braces attached to hooks at the front and back of the ring^45^. During the HV-aided walk test, the participants used a single-limb noninvasive ventilator (Trilogy100, Philips Respironics, Murrysville, Pennsylvania, USA), which was powered by an internal battery.

The ventilator settings were adjusted to prevent participants from rebreathing CO_2_. The support pressure levels in spontaneous timed mode were set to provide an inspiratory flow rate higher than 100 L/min^45^. The inspiratory and expiratory positive airway pressures were initially set to 16 and 6 cmH_2_O, respectively, and were then adjusted to remove any discomfort. To avoid patient–ventilator asynchrony and minimize breathing effort, the inspiratory rise time was set at level 1 and the ventilator off-cycling was set at 50% of the peak inspiratory flow rate^46^.

The helmet was removed immediately after the 6MWT. To ascertain the CO_2_ level inside the helmet for HV, the maximum CO_2_ tension was measured using a sampling line inserted 3 cm into the helmet through the sealed access ports. The maximum CO_2_ tension was recorded using a capnography monitor (Mindray Biological Medical Electronic Co, Ltd, Shenzhen, China) before and after the test (see Supplementary Fig. S2 online).

### Oxygen administration

Per ATS protocol, additional oxygen was administered if SpO_2_ was lower than 88%. Additional oxygen inflow was delivered through a traditional nasal cannula (flow rate = 3 L/min)^31^ or through connections with the helmet’s respiratory circuit (flow rate = 6 L/min).

### Experiment protocol

**Figure 2:**
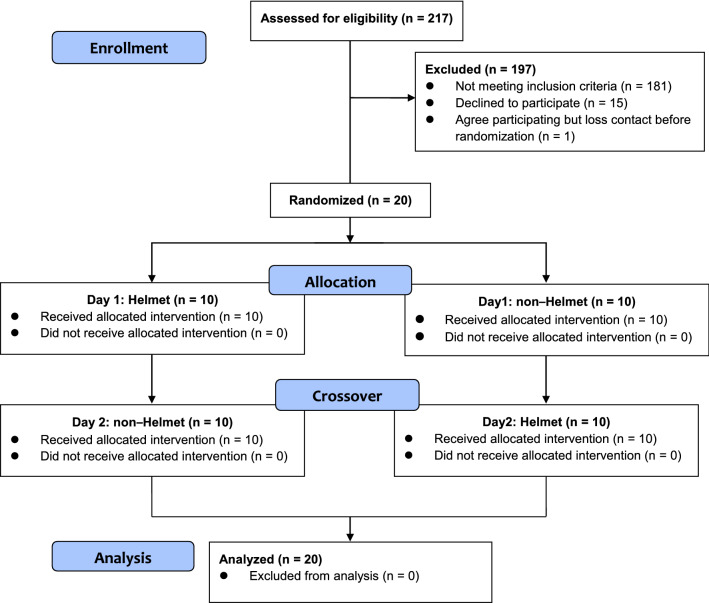
CONSORT flow diagram.

Two 6MWTs were performed with each participant in a randomized crossover design on two consecutive days (Fig. 2). The randomization sequence was performed on the http://randomization.com website. In the test in which HV was not provided, the participants underwent the 6MWT alone. The 6MWT was performed in accordance with the ATS guidelines^31^, and a checklist was used to report the study’s 6MWT design^47^. Because of the limitations of the experimental site, a straight, flat, 20-m-long corridor was used, which was shorter than the standard length in the ATS guidelines. Instructions were provided prior to the 6MWT to familiarize the participants with the 6MWT, and encouragement was provided during the test according to the ATS guidelines. All the 6MWTs were conducted by the same investigator. A trolley was used to carry the required devices, including the ventilator, oxygen cylinder, and monitors. Studies have indicated that if the patient uses a rollator or carries an oxygen cylinder, the accuracy of the 6MWD results can be affected^48,49^; thus, in this study, a research assistant pushed the trolley to avoid this problem.

### Outcome measurements

The primary outcome was the 6WMD, with or without HV. A wrist-worn pulse oximeter (WristOx2, Nonin Medical, Plymouth, Minnesota, USA) equipped with Bluetooth was sampled at 1 Hz with an averaging time of four beats. The WristOx2 data were automatically analyzed in Nonin nVision data management software (version 6.4) to reveal the HR and SpO_2_. A PtcCO_2_ monitor (TCM4, Radiometer, Medical AsP, Brønshøj, Denmark) was applied during the 6MWT to continually measure PtcCO_2_ using an electrochemical transducer. This was achieved by cleaning the measurement site with an alcohol pad, applying two or three drops of specific contact gel, and placing the sensor on the upper-left side of the chest. A noninvasive hemodynamic monitor that obtains measurements using electrical cardiometry (ICON, Osypka Medical, Berlin, Germany) was used before and immediately after the 6MWT. After the measurement site was cleaned with distilled water, an array of four surface electrocardiography electrodes was attached to the left side of the neck and the lower thorax (approximately at the level of the xiphoid process). The participants were asked to rate their dyspnea before and after the 6MWT. The self-report dyspnea score was assessed through a 0–10 modified Borg scale, with 0 indicating “none” and 10 indicating “the worst.” Baseline data were recorded for 30 min before the 6MWT, and outcome data (post 6MWT) were recorded immediately after the 6MWT. The EEI is calculated by dividing the mean 6MWT HR by the walking speed. In this study, a higher EEI represented poor walking economy^50^.

### Statistical analysis

Continuous data were presented as the mean ± standard deviation or median with interquartile range (IQR), depending on the distribution of the data (Shapiro–Wilk test). Categorical variables were compared using the exact McNemar test. Because the sample size was small, a Wilcoxon signed-rank test was used to analyze the continuous variables. Statistical analyses were performed using SPSS (version 22.0 for Windows, Chicago, Illinois, USA). Statistical significance was indicated at *p* < 0.05.

### Statement of ethics

We conducted the trial in accordance with good clinical practice guidelines and the Declaration of Helsinki. The study was approved by the Institutional Review Board for Human Studies of Fu Jen Catholic University, New Taipei City, Taiwan (C10832). Informed written consent was obtained from all participants.

## Supplementary Information

Supplementary Legends.

Supplementary Figure S1.

Supplementary Figure S2.

Supplementary Table S1.

Supplementary Table S2.

## Data Availability

The datasets used and/or analyzed during the current study are available from the first author on reasonable request.
