# Supplementary material for: A pilot crossover trial assessing the exercise performance patients with chronic obstructive pulmonary disease
Source: Sci Rep. 2022 Mar 9;12:4158. doi: 10.1038/s41598-022-07698-z (PMC8907196; doi:10.1038/s41598-022-07698-z)
Supplement: Supplementary file 1 — Supplementary Legends. [file 41598_2022_7698_MOESM1_ESM.docx]

**Supplementary legends**

Fig. S1. Detecting the maximum CO_2_ inside the helmet worn by a member of our research team.

Fig. S2. A member of our research team performing the 6-min walk test.
