# Supplementary figures and images for: A pilot crossover trial assessing the exercise performance patients with chronic obstructive pulmonary disease
Source: Sci Rep. 2022 Mar 9;12:4158. doi: 10.1038/s41598-022-07698-z (PMC8907196; doi:10.1038/s41598-022-07698-z)

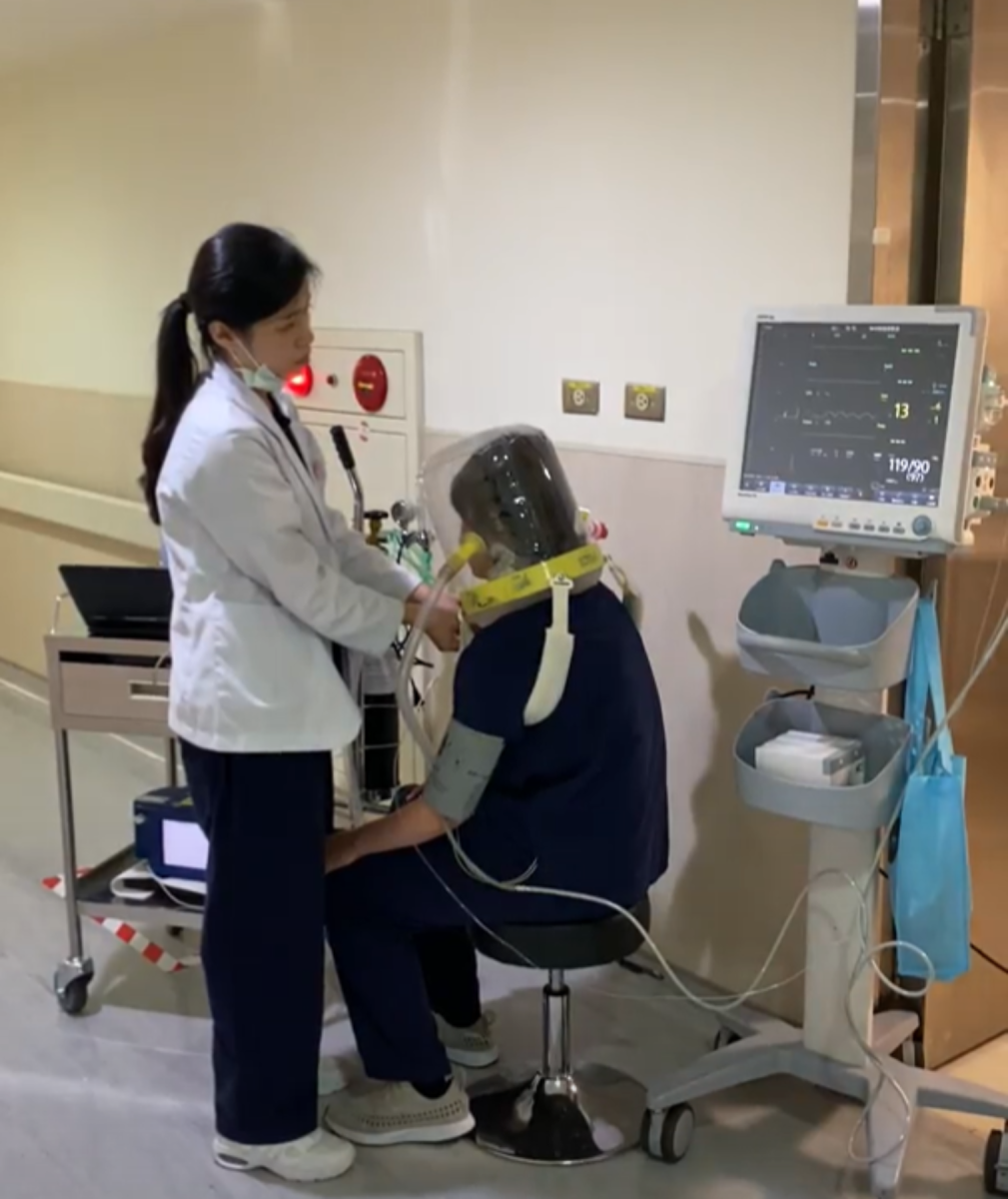

Supplement: Supplementary file 2 — Supplementary Figure S1. [file 41598_2022_7698_MOESM2_ESM.tiff]

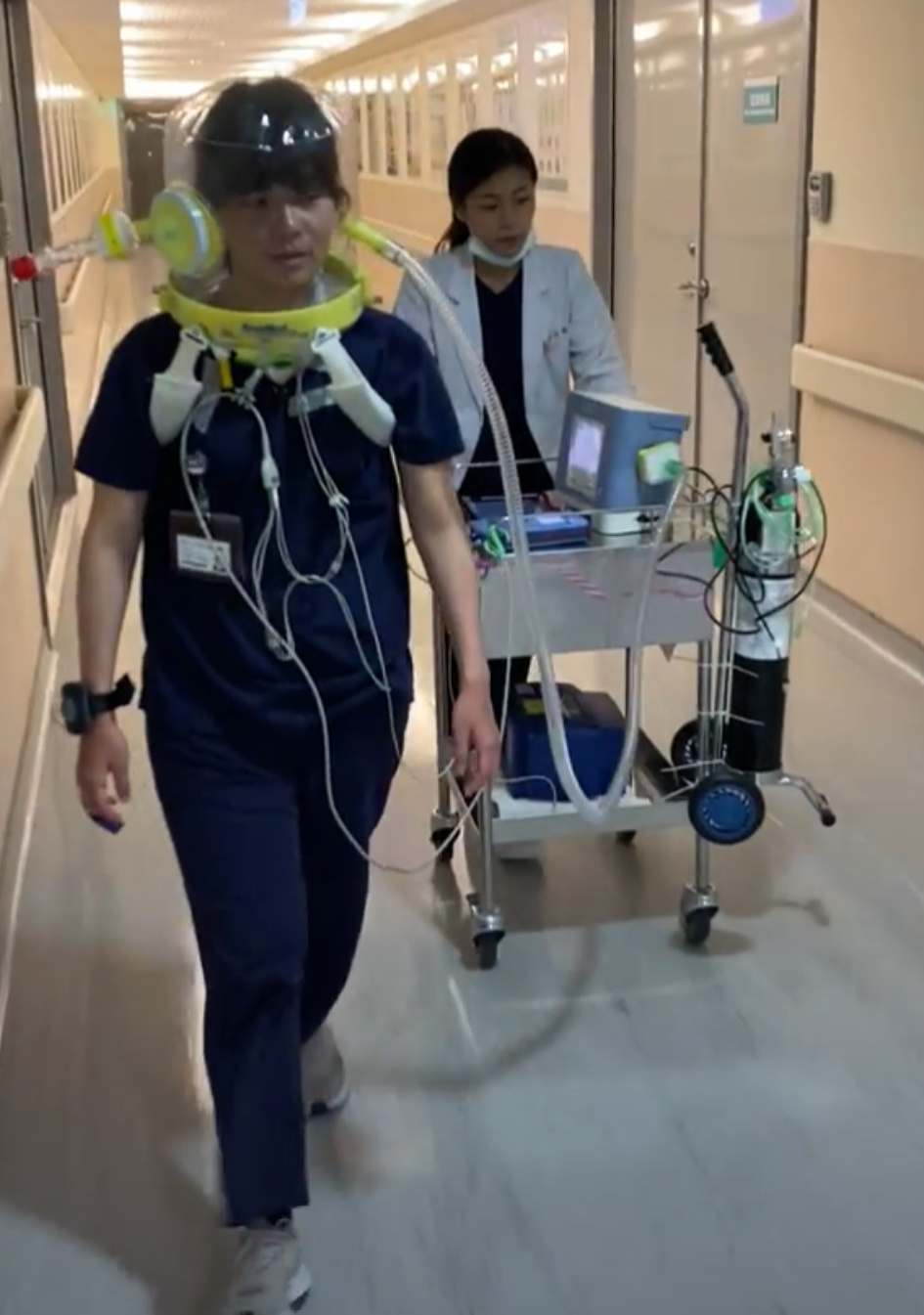

Supplement: Supplementary file 3 — Supplementary Figure S2. [file 41598_2022_7698_MOESM3_ESM.tiff]
