# Supplementary material for: A pilot crossover trial assessing the exercise performance patients with chronic obstructive pulmonary disease
Source: Sci Rep. 2022 Mar 9;12:4158. doi: 10.1038/s41598-022-07698-z (PMC8907196; doi:10.1038/s41598-022-07698-z)
Supplement: Supplementary file 4 — Supplementary Table S1. [file 41598_2022_7698_MOESM4_ESM.docx]

Table S1 Physiological and 6MWT Outcomes in Participants With Moderate Chronic Obstructive Pulmonary Disease

|  | **Helmet**  **Moderate stage**  **(n = 14)** | | **Non–Helmet**  **Moderate stage**  **(n = 14)** | | **Mean change**  **(Helmet minus non–Helmet)** | | | ***p*–value** |
| --- | --- | --- | --- | --- | --- | --- | --- | --- |
|  |  |  |  |  |  |  | **95% CI** |  |
| **6MWT outcome** |  |  |  |  |  |  |  |  |
| 6WMD, m | 325 | (312–337) | 312 | (265–355) | 19.5 | ±41.9 | -4.71–43.7 | 0.105 |
| Walking speed, m/min | 54.2 | (52–62.8) | 52 | (44.2–59.1) | 3.25 | ±6.99 | -0.78–7.28 | 0.105 |
| HR peak, b/m | 117 | (103–129) | 96 | (86–115) | 17 | ±11.1 | 10.6–23.4 | <0.001^***^ |
| SpO_2_ nadir, % | 91.5 | (89.3–93) | 92 | (89.8–93.3) | -0.29 | ±2.05 | -1.47–0.9 | 0.612 |
| EEI, beat/meter walked | 1.92 | (1.7–1.98) | 1.79 | (1.51–2.02) | 0.08 | ±0.39 | -0.15–0.3 | 0.473 |
| **Before 6MWT** |  |  |  |  |  |  |  |  |
| HR, b/m | 84.0 | (71–99.5) | 78.5 | (65.8–95.3) | 2.57 | ±7.06 | -1.50–6.65 | 0.196 |
| SpO_2_, % | 94.5 | (93.8–96.3) | 95 | (93.8–97) | -0.07 | ±1.69 | -1.04–0.9 | 0.876 |
| RR, b/m | 16 | (15–17) | 16 | (15–17) | 0 | ±0.55 | -0.32–0.32 | 1.000 |
| Borg-D | 0 | (0–1) | 0 | (0–2) | -0.43 | ±1.02 | -1.02–0.16 | 0.139 |
| sBP, mmHg | 131 | (117–139) | 127 | (120–138) | -1.50 | ±15.8 | -10.6–7.64 | 0.729 |
| dBP, mmHg | 80.5 | (64.8–85.3) | 79.5 | (68.8–87.8) | -1.29 | ±8.66 | -6.29–3.71 | 0.588 |
| MAP, mmHg | 95.7 | (84.1–102) | 95.7 | (87.8–104) | -8.60 | ±14.6 | -17.0–-0.18 | 0.046 |
| PtcCO_2_, mmHg | 42.5 | (39.8–44.3) | 44.5 | (39.8–46.3) | -2.29 | ±4.01 | -4.60–0.03 | 0.052 |
| **After 6MWT** |  |  |  |  |  |  |  |  |
| HR, b/m | 105 | (97.3–115) | 87.5 | (82.8–107) | 12.6 | ±9.57 | 7.05–18.1 | <0.001^***^ |
| SpO_2_, % | 92.5 | (91–93.3) | 94 | (93–5.3) | -0.93 | ±2.87 | -2.58–0.73 | 0.247 |
| RR, b/m | 20 | (19.8–23) | 18.5 | (18–19.3) | 2.07 | ±2.50 | 0.63–3.51 | 0.008^**^ |
| Borg-D | 3 | (1.75–5.25) | 3 | (1–4) | 0.64 | ±1.86 | -0.43–1.72 | 0.220 |
| sBP, mmHg | 153 | (137–165) | 139 | (125–157) | 14.7 | ±32.7 | -4.16–33.6 | 0.116 |
| dBP, mmHg | 82.5 | (73–87.3) | 87.5 | (70.8–95.5) | -2 | ±13.7 | -9.89–5.89 | 0.593 |
| MAP, mmHg | 106 | (94.5–115) | 103 | (91.3–117) | 3.57 | ±19.2 | -7.52–14.7 | 0.499 |
| PtcCO_2_, mmHg | 45 | (42.8–46.3) | 44 | (41–47.3) | 0.21 | ±4.84 | -2.58–3.01 | 0.871 |

Data are presented as mean ±SD or median (IQR). ^**^*p* <0.01; ^***^*p* <0.001.

6MWT: 6-minute walk test; 6WMD: 6-minute walk distance; HR: heart rate; SpO_2_: oxygen saturation; EEI: energy expenditure index; RR: respiratory rate; Borg-D: Borg dyspnea score; sBP: systolic blood pressure; dBP: diastolic blood pressure; MAP: mean arterial pressure; PtcCO_2_: transcutaneous carbon dioxide tension.
