# Supplementary material for: A pilot crossover trial assessing the exercise performance patients with chronic obstructive pulmonary disease
Source: Sci Rep. 2022 Mar 9;12:4158. doi: 10.1038/s41598-022-07698-z (PMC8907196; doi:10.1038/s41598-022-07698-z)
Supplement: Supplementary file 5 — Supplementary Table S2. [file 41598_2022_7698_MOESM5_ESM.docx]

Table S2 Physiological and 6MWT Outcomes in Participants with Mild Chronic Obstructive Pulmonary Disease

|  | **Helmet**  **Mild stage**  **(n = 6)** | | **Non–Helmet**  **Mild stage**  **(n = 6)** | | **Mean change**  **(Helmet minus non–Helmet)** | | | ***p*–value** |
| --- | --- | --- | --- | --- | --- | --- | --- | --- |
|  |  |  |  |  |  |  | **95% CI** |  |
| **6MWT outcome** |  |  |  |  |  |  |  |  |
| 6WMD, m | 418 | (354–439) | 404 | (350–452) | 5.83 | ±23.3 | -18.6–30.3 | 0.567 |
| Walking speed, m/min | 69.7 | (59–73.2) | 67.3 | (58.4–75.3) | 0.97 | ±3.89 | -3.11–5.05 | 0.567 |
| HR peak, b/m | 110 | (95.3–118) | 107 | (94.0–110) | 2.83 | ±15.7 | -13.7–19.3 | 0.677 |
| SpO_2_ nadir, % | 93.5 | (88.5–94.5) | 93.5 | (89.8–95) | -1.50 | ±6.09 | -7.89–4.89 | 0.573 |
| EEI, beat/meter walked | 1.46 | (1.35–1.77) | 1.48 | (1.24–1.71) | 0.03 | ±0.25 | -0.24–0.3 | 0.797 |
| **Before 6MWT** |  |  |  |  |  |  |  |  |
| HR, b/m | 86 | (68.5–106) | 88 | (74.3–104) | -1.33 | ±7.71 | -9.43–6.76 | 0.690 |
| SpO_2_, % | 96 | (95–98) | 97.5 | (97–98) | -1.17 | ±1.17 | -2.39–0.06 | 0.058 |
| RR, b/m | 16 | (14.8–17.3) | 15.5 | (14.8–16) | 0.67 | ±0.82 | -0.19–1.52 | 0.102 |
| Borg-D | 0 | (0–0.75) | 0 | (0–0) | 0.50 | ±1.22 | -0.79–1.79 | 0.363 |
| sBP, mmHg | 122 | (100–129) | 123 | (112–138) | -7.33 | ±19.4 | -27.7–13.1 | 0.398 |
| dBP, mmHg | 82 | (65.3–87) | 78 | (61.3–83.8) | 2.83 | ±4.62 | -2.02–7.68 | 0.194 |
| MAP, mmHg | 95.8 | (76.9–102) | 92.8 | (84–98.9) | -0.56 | ±7.06 | -7.96–6.85 | 0.855 |
| PtcCO_2_, mmHg | 37.5 | (35.8–40.8) | 41 | (38–44) | -2.50 | ±4.46 | -7.18–2.18 | 0.228 |
| **After 6MWT** |  |  |  |  |  |  |  |  |
| HR, b/m | 102 | (89.5–118) | 98.5 | (92.3–108) | 1.33 | ±19.1 | -18.7–21.3 | 0.871 |
| SpO_2_, % | 94.5 | (94–96.3) | 94 | (92–96.3) | 1.17 | ±3.97 | -3.00–5.33 | 0.504 |
| RR, b/m | 18.5 | (17–22) | 17.5 | (17–18.5) | 1.33 | ±2.42 | -1.21–3.88 | 0.235 |
| Borg-D | 2 | (0–5) | 0.5 | (0–0) | 1.33 | ±2.34 | -1.12–3.79 | 0.221 |
| sBP, mmHg | 150 | (104–167) | 147 | (134–157) | -3.50 | ±32.4 | -37.5–30.5 | 0.802 |
| dBP, mmHg | 91 | (69.3–98.5) | 82 | (69.3–93.3) | 4.83 | ±6.15 | -1.62–11.3 | 0.112 |
| MAP, mmHg | 113 | (80.7–118) | 103 | (90.3–113) | 2.06 | ±13.5 | -12.1–16.2 | 0.725 |
| PtcCO_2_, mmHg | 41 | (39–46) | 41.5 | (37.3–45.3) | 1.50 | ±4.85 | -3.59–6.59 | 0.483 |

Data are presented as mean ±SD or median (IQR). ^**^*p* <0.01; ^***^*p* <0.001.

6MWT: 6-minute walk test; 6WMD: 6-minute walk distance; HR: heart rate; SpO_2_: oxygen saturation; EEI: energy expenditure index; RR: respiratory rate; Borg-D: Borg dyspnea score; sBP: systolic blood pressure; dBP: diastolic blood pressure; MAP: mean arterial pressure; PtcCO_2_: transcutaneous carbon dioxide tension.
